# Supplementary figures and images for: Chitosan-DNA nanoparticles: synthesis and optimization for long-term storage and effective delivery
Source: PeerJ. 2025 Jan 24;13:e18750. doi: 10.7717/peerj.18750 (PMC11771301; doi:10.7717/peerj.18750)

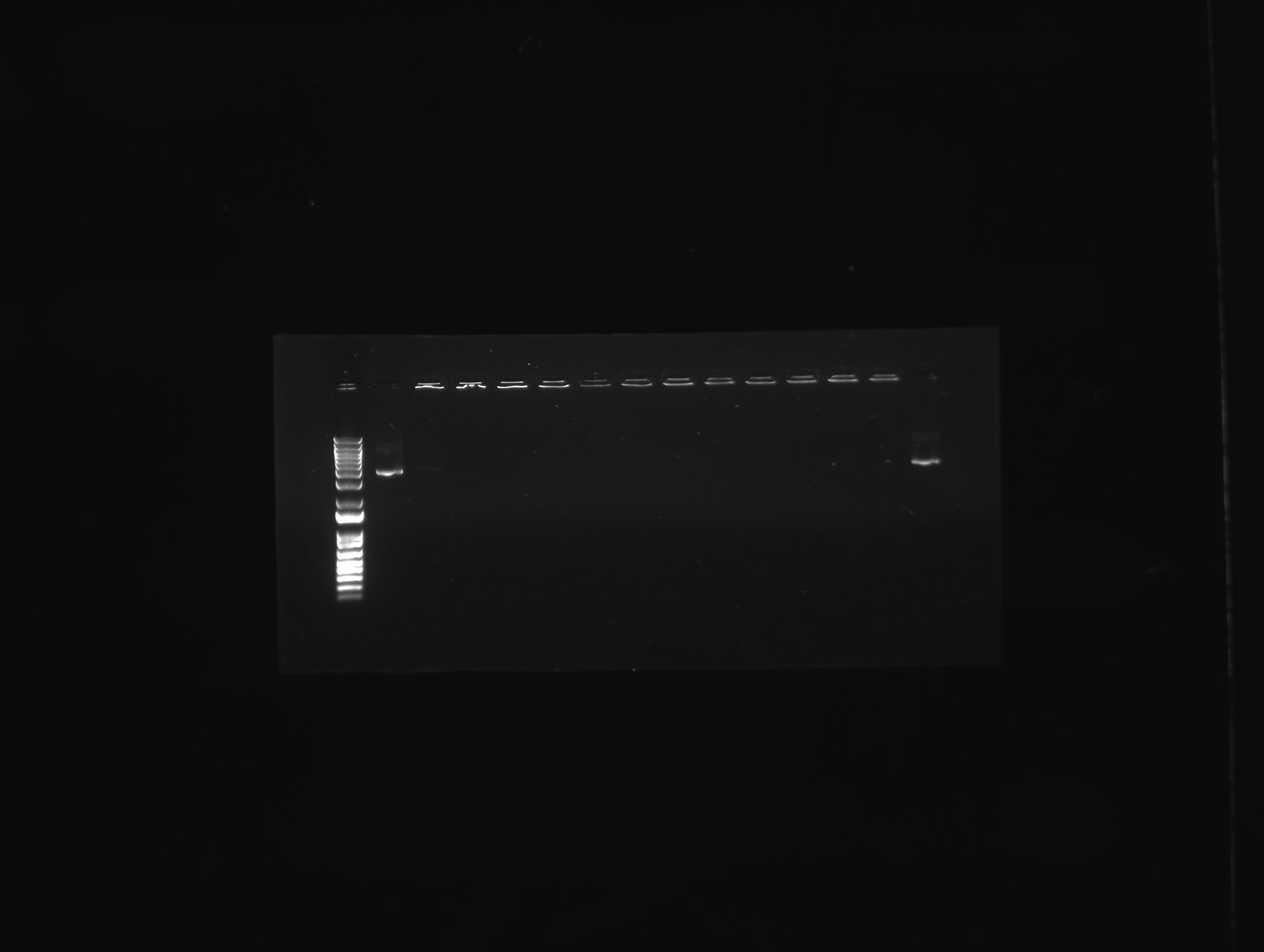

Supplement: Supplemental Information 2 [file peerj-13-18750-s002.zip › Original gel/24 hours gel.jpg]

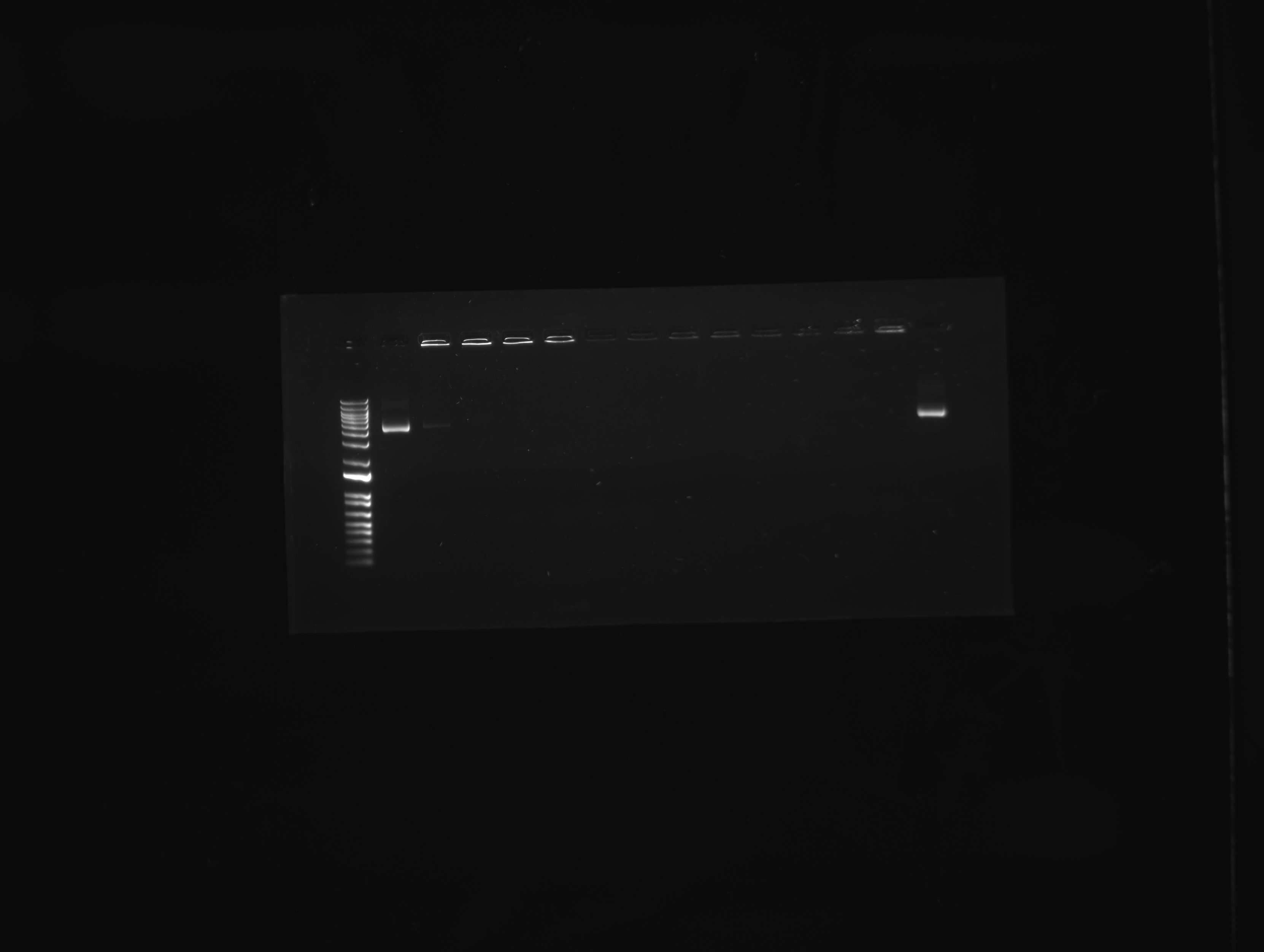

Supplement: Supplemental Information 2 [file peerj-13-18750-s002.zip › Original gel/48 hours gel.jpg]

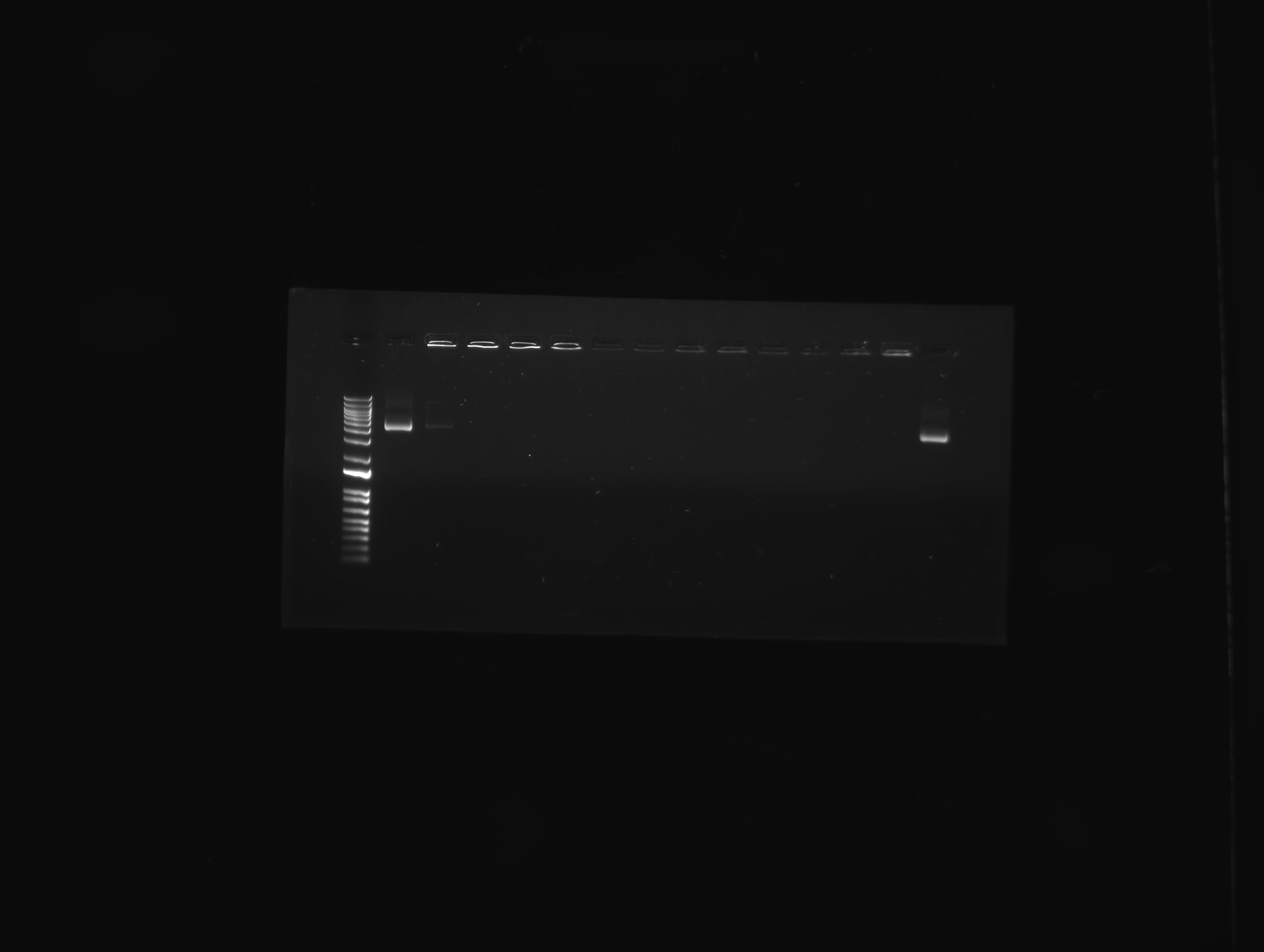

Supplement: Supplemental Information 2 [file peerj-13-18750-s002.zip › Original gel/72 hours gel.jpg]

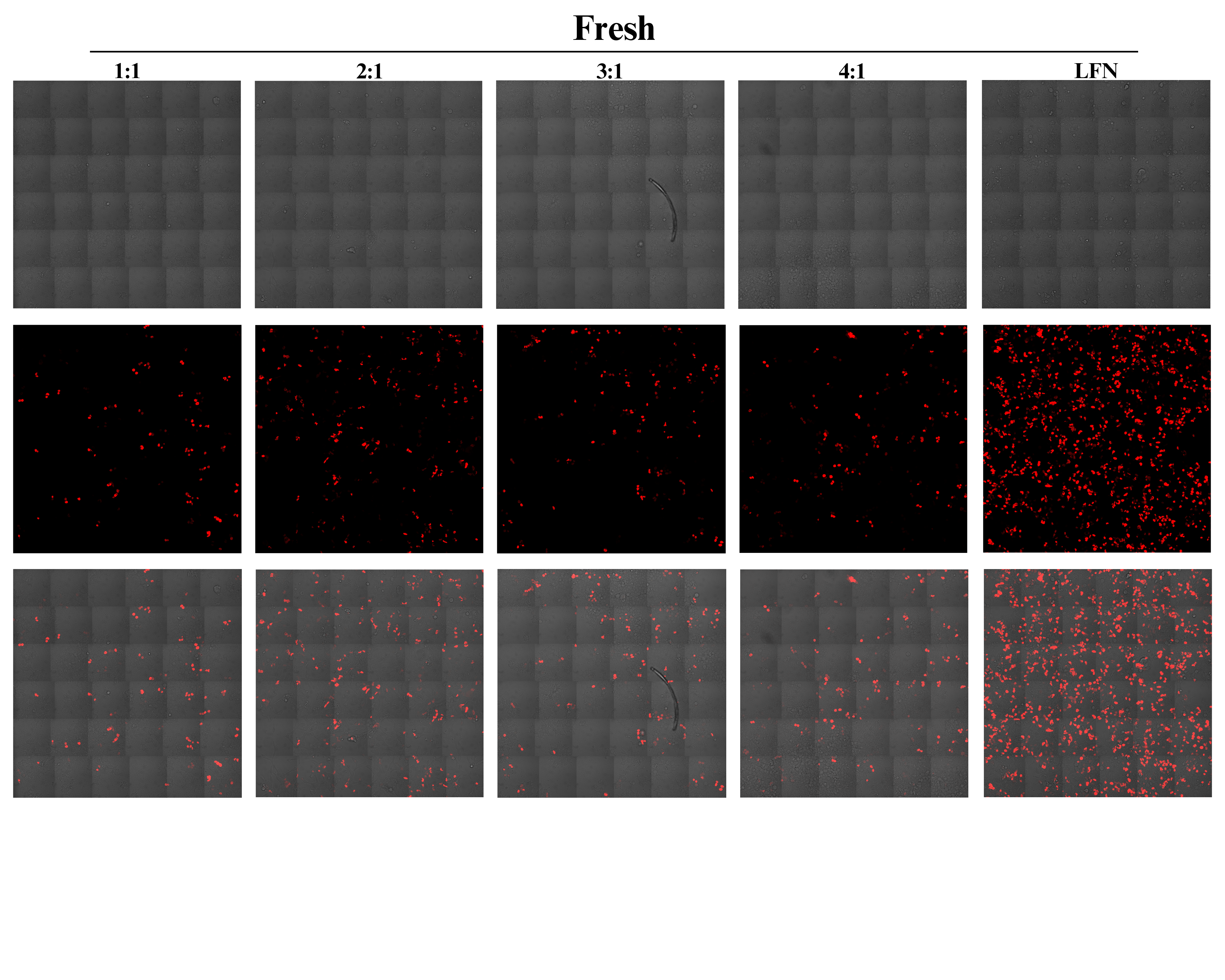

Supplement: Supplemental Information 4 — LFN stands for Lipofectamine, -ve stands for negative control. [file peerj-13-18750-s004.png]

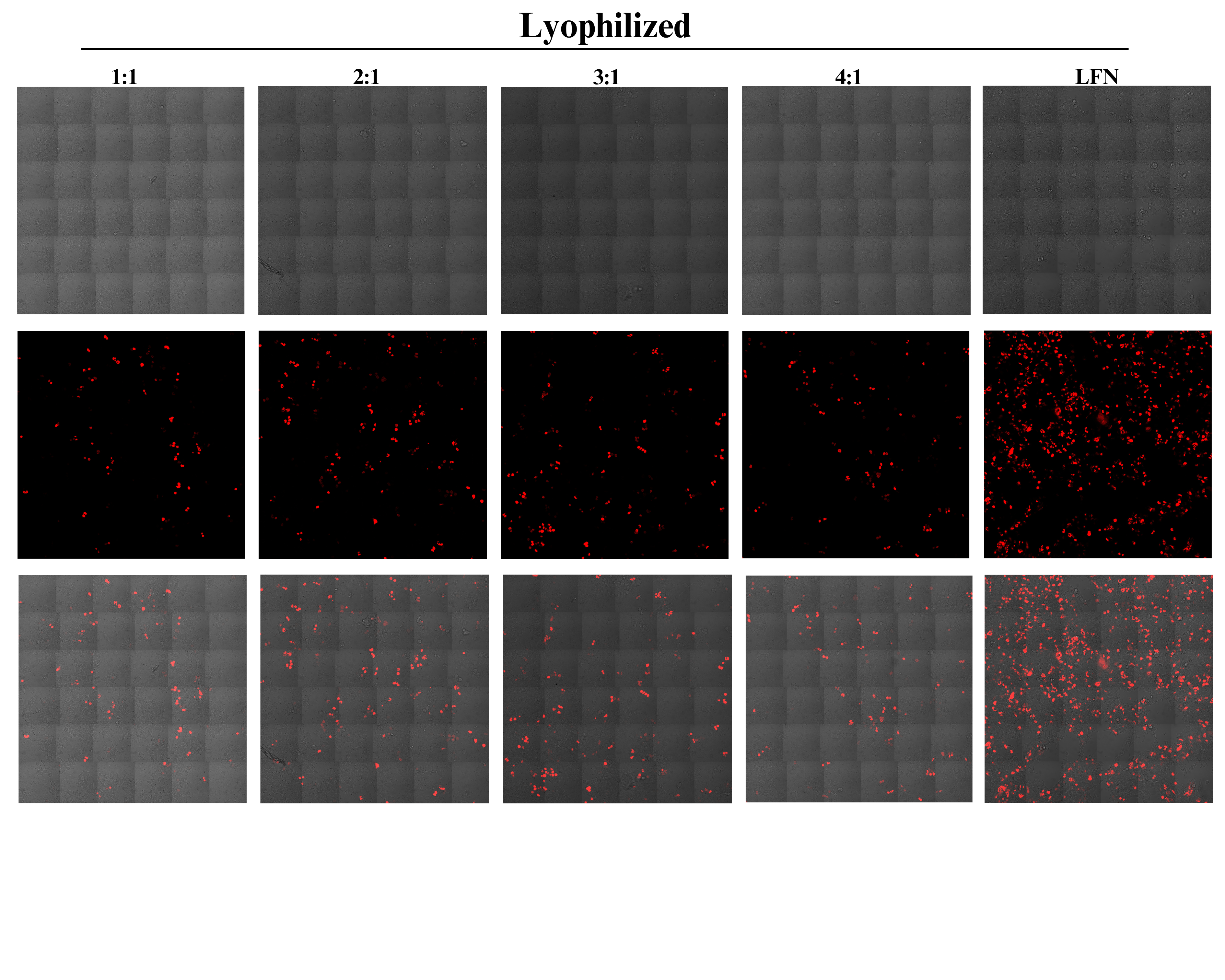

Supplement: Supplemental Information 5 — LFN stands for Lipofectamine, -ve stands for negative control. [file peerj-13-18750-s005.png]
