# Supplementary material for: Chitosan-DNA nanoparticles: synthesis and optimization for long-term storage and effective delivery
Source: PeerJ. 2025 Jan 24;13:e18750. doi: 10.7717/peerj.18750 (PMC11771301; doi:10.7717/peerj.18750)
Supplement: Supplemental Information 3 — Different ratios (1:1-4:1) of CsNPs were treated with DMEM for 24, 48 and 72 hours as mean (n=5) ±SD. During the time and different ratios, the mean size of the particles was not changed much, indicating CsNPs were stable during the time. [file peerj-13-18750-s003.docx]

**Supplementary Table 1**

| **Incubation time (h)** |  | **CsNPs ratio** | **Mean size (nm)±SD** | **Zeta potential (mV)±SD** |
| --- | --- | --- | --- | --- |
| **24 hours** | **Fresh** | 1:1 | 234.8±16.0 | 10.910±0.438 |
|  |  | 2:1 | 288.6±18.3 | 18.608±1.276 |
|  |  | 3:1 | 370.1±17.3 | 30.910±0.438 |
|  |  | 4:1 | 399.1±24.4 | 33.821±0.568 |
|  | **Lyophilized sonicated** | 1:1 | 246.4±11.1 | 11.042±0.375 |
|  |  | 2:1 | 302.0±35.8 | 19.443±0.759 |
|  |  | 3:1 | 358.3±25.0 | 30.768±0.324 |
|  |  | 4:1 | 396.0±23.3 | 34.970±0.295 |
| **48 hours** | **Fresh** | 1:1 | 296.6±12.7 | 10.845±0.296 |
|  |  | 2:1 | 313.8±10.3 | 20.250±1.464 |
|  |  | 3:1 | 389.8±17.3 | 30.828±0.370 |
|  |  | 4:1 | 394.4±27.1 | 33.529±0.839 |
|  | **Lyophilized sonicated** | 1:1 | 270.6±5.4 | 11.237±0.407 |
|  |  | 2:1 | 305.2±4.0 | 19.076±0.782 |
|  |  | 3:1 | 360.6±17.9 | 31.306±0.398 |
|  |  | 4:1 | 407.1±17.6 | 33.993±0.463 |
| **72 hours** | **Fresh** | 1:1 | 300.4±11.1 | 11.415±1.025 |
|  |  | 2:1 | 315.6±8.6 | 21.336±1.021 |
|  |  | 3:1 | 379.0±37.1 | 32.190±1.999 |
|  |  | 4:1 | 397.8±8.6 | 33.360±0.753 |
|  | **Lyophilized sonicated** | 1:1 | 278.6±7.2 | 11.145±0.425 |
|  |  | 2:1 | 309.1±5.8 | 19.790±0.502 |
|  |  | 3:1 | 349.6±11.4 | 30.945±0.485 |
|  |  | 4:1 | 408.4±5.5 | 33.901±0.429 |
