# Supplementary material for: Chitosan-DNA nanoparticles: synthesis and optimization for long-term storage and effective delivery
Source: PeerJ. 2025 Jan 24;13:e18750. doi: 10.7717/peerj.18750 (PMC11771301; doi:10.7717/peerj.18750)
Supplement: Supplemental Information 7 [file peerj-13-18750-s007.docx]

Links to Flow cytometry results here in Zenodo:

DOI of Figure 3: [10.5281/zenodo.13709923](https://doi.org/10.5281/zenodo.13709923)

DOI of Figure 4: [10.5281/zenodo.13710021](https://doi.org/10.5281/zenodo.13710021)

DOI of Figure 5: [10.5281/zenodo.13710101](https://doi.org/10.5281/zenodo.13710101)
